# Supplementary material for: Supply-side options to reduce land requirements of fully renewable electricity in Europe
Source: PLoS One. 2020 Aug 6;15(8):e0236958. doi: 10.1371/journal.pone.0236958 (PMC7410258; doi:10.1371/journal.pone.0236958)
Supplement: S1 File — (PDF) [file pone.0236958.s001.pdf]

Supply-side options to reduce land requirements of fully renewable electricity in Europe

S1 Supporting Information

Tim Tröndle

2020-07-23

1 Impact of assuming no further hydroelectricity capacity expansion

In this study, I do not consider any expansion of hydroelectricity capacities from today's levels. This is a slightly conservative assumption, as Gernaat et al. [1] find that Europe's hydroelectricity potential is 50% above today's generation when considering technical, economic, and ecological constraints. However, the authors find that 85% of this expansion stems from run-of-river installations whose land requirements are small. A significant land use change due to expansion of hydro electricity is therefore unlikely in Europe.

Any expansion of dispatchable hydroelectricity capacity likely impacts my results, because such capacity can provide balancing and reduce the need for other – likely more costly – forms of balancing. I expect higher shares of dispatchable hydroelectricity capacity to reduce total system cost especially of the solar-centred cases. However, according to Gernaat et al. [1] the potential to expand this kind of capacity is low (< 1% of current electricity demand of the European Union) and thus I consider the impact of further capacity expansion of dispatchable capacity on the results of this study to be low.

2 Map of land requirements

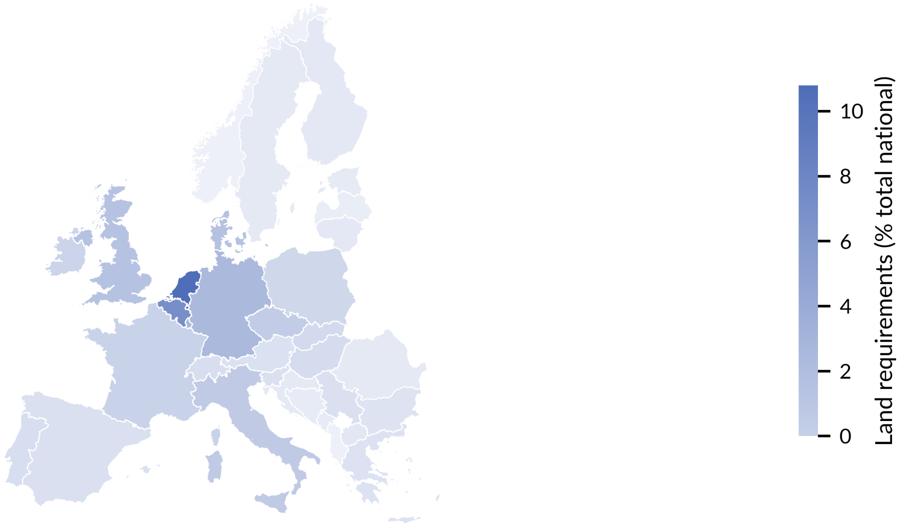

Fig S1: Expected land requirements of the case with minimal expected total system cost. In the case with minimal expected cost, each country is supplied to 50% from each onshore wind and utility-scale PV next to hydroelectricity of today's levels. Land requirements are given relative to total national land.

3 Expected land requirements when spacing of wind power is ignored

In the estimate of land requirements of onshore wind turbines applied in this study, I include the spacing between wind turbines. While the spacing does allow for a dual use of the land together with agriculture, it excludes other land uses and it adds to the visual impact of wind power. For these reasons, land requirements of wind power in this study do not only comprise the land used for the actual towers, access roads, and auxiliary infrastructure, but also the spacing between the wind turbines.

However, spacing makes up the largest part of the land requirements of wind turbines. For applications in which other impacts of renewable infrastructure are in focus, for example the sealing of land, one may want to exclude spacing from wind power's land requirements. Without spacing, wind power has a capacity density of 263 W/m^2 [2] which is very high and, in fact, even higher than the one of solar power. Consequently, when aiming at minimising direct land requirements stemming from infrastructure only, onshore wind power is an attractive supply-side option, see Fig S2. Wind power has a large visual impact on landscapes, potentially devaluing land and excluding other uses, but its local land footprint is small.

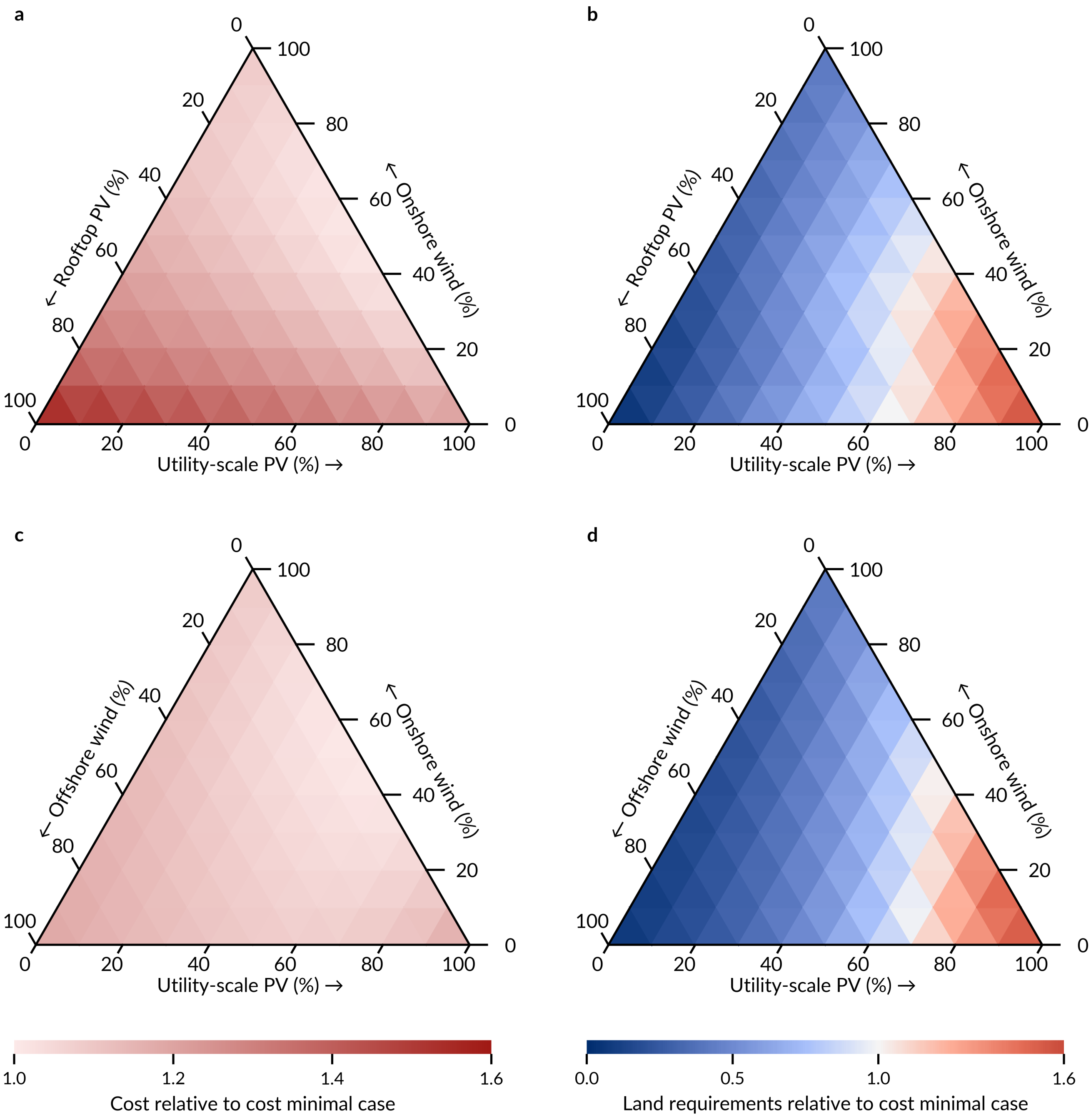

Fig S2: Expected cost and land requirements of fully renewable electricity systems with all possible shares of three different supply technologies when spacing of onshore wind turbines is ignored. All cases include hydroelectricity of today's capacity and bioenergy from residuals next to three solar and wind technologies. Expected values are the means of uncertainty distributions. **a,b,** Total system cost (**a**) and land requirements (**b**) of cases with utility-scale PV, onshore wind and rooftop PV as supply side options. **c,d,** Total system cost (**c**) and land requirements (**d**) of cases with utility-scale PV, onshore wind, and offshore wind as supply side options.

References

1. Gernaat DEHJ, Bogaart PW, Vuuren DP van, Biemans H, Niessink R. High-resolution assessment of global technical and economic hydropower potential. Nat Energy. 2017;2: 821–828. doi:10.1038/s41560-017-0006-y

2. Palmer-Wilson K, Donald J, Robertson B, Lyseng B, Keller V, Fowler M, et al. Impact of land requirements on electricity system decarbonisation pathways. Energy Policy. 2019;129: 193–205. doi:10.1016/j.enpol.2019.01.071
