# Supplementary material for: Supply-side options to reduce land requirements of fully renewable electricity in Europe
Source: PLoS One. 2020 Aug 6;15(8):e0236958. doi: 10.1371/journal.pone.0236958 (PMC7410258; doi:10.1371/journal.pone.0236958)
Supplement: S1 Code — (ZIP) [file pone.0236958.s003.zip › code/report/template.html]

$for(author-meta)$
$endfor$
$if(date-meta)$
$endif$
$if(keywords)$
$endif$
$if(title-prefix)$$title-prefix$ – $endif$$pagetitle$
$if(highlighting-css)$
$endif$
$for(css)$
$endfor$
$if(math)$
$math$
$endif$
$for(header-includes)$
$header-includes$
$endfor$

$for(include-before)$
$include-before$
$endfor$
$if(title)$

# $title$

$if(subtitle)$

$subtitle$

$endif$
$for(author)$

$author$

$endfor$
$if(date)$

$date$

$endif$
$if(publication)$

The original version of this article has been published in $publication.journal$.

$endif$
$if(highlights)$

# Highlights

$for(highlights)$- $highlights$
$endfor$
$endif$
$if(abstract)$

# Abstract

$abstract$
$endif$
$endif$
$if(toc)$
$table-of-contents$
$endif$
$body$
$for(include-after)$
$include-after$
$endfor$
